# Comment on: the myth of pulmonary metastasectomy

**DOI:** 10.1038/s41416-020-01061-7

**Published:** 2020-09-22

**Authors:** Matthieu Zellweger, Michel Gonzalez

**Affiliations:** Service of Thoracic Surgery, University Hospital of Lausanne, Lausanne, Switzerland

**Keywords:** Surgical oncology, Metastasis

We read with great interest the comment entitled “The myth of pulmonary metastasectomy”.^1^ Macbeth and co-workers^2^ question the benefit of pulmonary metastasectomy and refer to recent results showing no statistical difference in 5-year survival between patients undergoing surgical metastasectomy and control patients.

The randomised PulmiCC trial included 30–35 patients in each arm and was aborted due to insufficient recruitment. One reason was that many patients chose to undergo surgery. Furthermore, more than twice as many patients were assigned to surgical management because the clinical team overrode the study protocol. This is likely rooted in surgical practice: we observe daily that single or multiple pulmonary metastasectomies may result in long-term survival or even cure.

We agree that the survival benefit for surgical patients is probably modest. Exactly how modest is a question that might never be answered: many sources report low survival rates for untreated patients with LMs following CRC. On the other hand, the authors of the PulmiCC trial report very good survival rates in control patients, albeit with a non-significant tendency to be slightly shorter than in surgical patients. However, the PulmiCC trial control group was by no means a group of untreated patients. They were certainly followed very closely and very carefully. Hence, these results should be considered the very best survival rate that can be achieved without surgery, and not the crude survival rate of untreated patients, a figure frequently quoted as the (possibly exaggerated) lower baseline against which to measure any survival improvement offered by a treatment modality.

Many metastatic cancer patients cling to the hope to improve a seemingly desperate situation. We think that survival benefit is only one among many aspects that should be discussed before pulmonary metastasectomy.

These patients should be discussed in multidisciplinary settings (including surgeon, oncologist, radio-oncologist, radiologist, pathologist, pulmonologist) to propose the most appropriate treatment. Surgery might not always be the optimal choice, depending on clinical aspects (number of lesions, localisation, general status). Nowadays, surgical resections can be accomplished by VATS with short hospitalisation stays and virtually no morbidity and mortality.^3^ This is the preferred approach for solitary pulmonary nodules. It allows complete resections and helps confirm the diagnosis of metastasis. In our experience, only 50% of cancer patients with a solitary nodule actually have a metastasis.^4^ Clearly, misdiagnosed conditions might result in a wrong subsequent treatment and only surgery can ascertain some diagnostic elements. In addition, tissue samples collected during surgery provide the possibility to analyse prognostic biomarkers, make further research and propose targeted and individualised oncological treatments.^3^

We fully agree with Macbeth et al. that surgery should not be offered as a one-size-fits-all management for all metastatic patients. We do however wish to stress that as of today, whereas it is certain that some patients will really benefit from curative surgical management, it is not entirely clear which ones.^5,6^

We wish to conclude in more general terms. Many patients entertain the hope that the best possible treatment is to remove any trace of malignancy from their body. This is not entirely unfounded. We, as surgeons, understand that the likelihood of cure might not be 100%, or not even near, but we struggle to predict who will benefit from surgical resection. Although the probability to cure might be low, we refuse to deny it to our patients. We always balance the risks and benefits of surgery and of chemotherapy. Let us only state that chemotherapy is no walk in the park.

Randomised trials are crucial in determining the best treatment options. In agreement with Macbeth et al., we regret that they are few and far between. Although these might question the current views that surgical resection offers a real survival benefit, we wish to stress that the patients’ hope to be cured, compounded by our inability to predict if this will be the case, is the most difficult aspect to study in a randomised clinical trial.

In daily practice, we discuss the expectations and anxieties of cancer patients who want to be treated. We must acknowledge that of the two evils (live with the uncertainty that cancer might recur after chemotherapy or derive uncertain benefits from a potentially curative surgery and confirm the diagnosis), each patient must choose which one they perceive as the lesser. This dilemma might explain the low recruitment of the PulmiCC trial. Finally, as doctors we want the best for our patients. In that sense, we experience daily that surgery can alleviate uncertainties and may even give hope.

## Data Availability

Not applicable.
